# Evaluation of Anti-SARS-CoV-2 IgA Response in Tears of Vaccinated COVID-19 Subjects

**DOI:** 10.3390/v15020399

**Published:** 2023-01-30

**Authors:** Irene Soffritti, Maria D’Accolti, Carla Gallenga, Roberto De Giorgio, Matteo Guarino, Martina Maritati, Francesca Bini, Eleonora Mazziga, Carlo Contini, Elisabetta Caselli

**Affiliations:** 1 Department of Chemical, Pharmaceutical, and Agricultural Sciences, and LTTA, Section of Microbiology, University of Ferrara, 44121 Ferrara, Italy; 2 Infectious Diseases Unit, Department of Medical Sciences, University of Ferrara, 44121 Ferrara, Italy; 3 Department of Translational Medicine, University of Ferrara, 44121 Ferrara, Italy

**Keywords:** IgA, ocular mucosal immunity, COVID-19 vaccine

## Abstract

Secretory IgA (sIgA), which may play an important role in the early defense against SARS-CoV-2 infection, were detected in the eye of COVID-19 patients. However, an evaluation of the sIgA response in the tears of vaccinated or non-vaccinated COVID-19 subjects is still lacking. Aimed at characterizing sIgA mucosal immunity in the eye, this study analyzed tear samples from 77 COVID-19 patients, including 63 vaccinated and 14 non-vaccinated subjects. The groups showed similar epidemiological features, but as expected, differences were observed in the percentage of asymptomatic/pauci-symptomatic subjects in the vaccinated vs. non-vaccinated cohort (46% and 29% of the total, respectively). Consistent with this, ocular sIgA values, evaluated by a specific quantitative ELISA assay, were remarkably different in vaccinated vs. non-vaccinated group for both frequency (69.8% vs. 57.1%, respectively) and titer (1372.3 U/mL vs. 143.7 U/mL, respectively; *p* = 0.01), which was significantly differently elevated depending on the type of administered vaccine. The data show for the first time significant differences of available vaccines to elicit sIgA response in the eye and suggest that quantitative tear-based sIgA tests may potentially serve as a rapid and easily accessible biomarker for the assessment of the development of a protective mucosal immunity toward SARS-CoV-2.

## 1. Introduction

The severe acute respiratory syndrome coronavirus 2 (SARS-CoV-2), responsible for the COVID-19 pandemic, is primarily spread by airborne transmission, and several pieces of evidence highlight the role of the conjunctiva in virus entry, acquisition, and transmission [1]. The ocular surface, composed by epithelial cells of the conjunctiva and cornea, is constantly exposed to respiratory droplets and aerosol and could be the site of virus entry through interaction with the angiotensin-converting enzyme 2 (ACE2) receptors and transmembrane protease serine 2 (TMPRSS2) cofactors, which have been found highly expressed on the human ocular surface [2,3]. In addition, the transfer of the virus through the nasolacrimal duct to the upper airways has been suggested as a possible transmission onset [4].

Confirming these observations, the use of eye glasses in the general population and specific eye protection among community health workers has been associated with a decrease of SARS-CoV-2 acquisition, supporting that the ocular mucosal surface may be considered an important route of transmission [5,6,7].

The presence of SARS-CoV-2 RNA has been detected by molecular methods (polymerase chain reaction after reverse transcription, RT-PCR) in tears and conjunctival fluid of infected subjects although with low levels and variable positivity [8,9,10]. 

Besides these findings regarding the viral presence and replication in the eye, relatively little is known about the acquisition and development of mucosal immunity specifically directed against SARS-CoV-2 at the ocular surface. The surface of the eye represents a fundamental protective barrier against the invasion of several pathogens through different protective mechanisms involving lactoferrin, lysozyme, lipocalin, and immunoglobulin IgA [10]. 

SARS-CoV-2 infection is able to trigger both systemic and mucosal immune responses, inducing the production of serum IgG and secretory IgA (sIgA) in mucosal secretions including saliva, nasal fluid, and tears [11,12,13]. SARS-CoV-2 specific IgA has been demonstrated to effectively neutralize the virus at the mucosal surfaces, binding the viral spike protein, thereby blocking its interaction with the host ACE2 receptor [14].

A prolonged presence of anti-SARS-CoV-2 sIgA in tears has been previously observed by our group in about 36% of COVID-19 patients, persisting up to 48 days post disease onset [11]. More recently, the sIgA presence in tears was confirmed in around 45% of COVID-19 patients, correlating with high IgA positivity at the serum level although no correlation was observed between IgA antibody level in tear fluid and disease stage [8]. Based on these observations, the evaluation of sIgA in tears of COVID-19 patients has been suggested as a potential biomarker for the detection of the development of individual host mucosal immunity to SARS-CoV-2 infection [8]. 

On the other hand, COVID-19 vaccination, comprising the administration of two or three doses of vaccine, proved to be highly protective from SARS-CoV-2 infection, being able to stimulate the systemic antibody and cellular immune response [15,16,17]. However, data regarding the effect of vaccination in inducing a mucosal sIgA response are very limited, and conclusive results are still lacking. A recent study by Lambiase et al. evidenced that the administration of the third dose of vaccine (booster dose) is able to stimulate a recovery of humoral response but shows a limited activity in stimulating mucosal immune response in comparison to prior SARS-CoV-2 infection [17].

To our knowledge, there are no available results regarding the state of the local mucosal response at the ocular level in vaccinated individuals who subsequently developed COVID-19. The aim of this study was to analyze the presence and amount of sIgA in the conjunctival fluid of COVID-19 patients by comparing SARS-CoV-2 vaccinated and non-vaccinated subjects.

## 2. Materials and Methods

### 2.1. Subjects and Study Design

An observational study was conducted at the COVID Unit of the University Hospital of Ferrara (Italy) to assess the presence of secreted anti-SARS-CoV-2 IgA at the eye level in vaccinated or non-vaccinated subjects infected by SARS-CoV-2. Prior to subjects recruitment, the study was approved by the local Ethics Committee (Code ID. 544/2020/Oss/AOUFe, approved on 11/06/2020), and all enrolled recruits provided an informed consent. To assess the presence and amount of IgA response in the ocular fluids, a total of seventy-seven subjects were recruited, who were positive for SARS-CoV-2 RNA presence at the time of enrollment as judged by nasopharyngeal/oropharyngeal swabs (NPS/OPS) and molecular analysis by polymerase chain reaction after reverse transcription (RT-PCR), performed by the central unique hospital microbiology unit by using the Alinity m Resp-4-Plex AMP kit (Abbott Molecular, Chicago, lL, USA). Of them, 63 subjects had received at least two doses of anti-SARS-CoV-2 vaccine prior to hospitalization. Administered vaccines included RNA-based monovalent vaccines Comirnaty (Pfizer-BioNTech) and Spikevax (Moderna), whereas only one patient had received vaccination with the recombinant adenovirus-based Vaxzevria vaccine (AstraZeneca). The non-vaccinated group included 14 subjects who did not receive any type of SARS-CoV-2 vaccination. The presence of specific ocular and general COVID-19-related symptoms was recorded as well as the subjects’ epidemiological features (age, gender) and general parameters including blood lymphocyte count, steroid therapy, and C-reactive protein (CRP) concentration. The time between first observation and sampling was also recorded.

### 2.2. Sample Collection

Tear samples were collected from Infectious Diseases and Internal Medicine of the University Hospital of Ferrara from both eyes by Schirmer test strip, as previously described [11,18]. Immediately after sampling, the strips were immersed in 0.4 mL of sterile phosphate buffer (PBS), refrigerated (2–8 °C), and transported within 2 h to the microbiology laboratory, where samples were vortexed 30 sec and collected by centrifugation, then stored at –80 °C until use. The study was approved by the local Ethics Committee of the St. Anna Hospital. Each enrolled subject signed an informed consent before recruitment.

### 2.3. IgA Analysis

The evaluation of anti-SARS-CoV-2 IgA levels in the tears collected from vaccinated and unvaccinated COVID-19 patients was performed by the quantitative ELISA assay RayBio COVID-19 S1RBD protein Human IgA ELISA Kit (IEQ-CoVS1RBD-IgA; RayBiotech Life, Peachtree Corners, GA, United States), targeting human IgA directed against the receptor-binding domain of the viral spike protein S1-RBD. This COVID-19 human IgA antibody ELISA kit employs an indirect ELISA method, with standard 96-well plates coated with the SARS-CoV-2 S1-RBD protein. A 1000 Unit/ml (U/mL) positive control (provided with the kit) is used to prepare a standard eight-point calibration curve (1000, 333.3, 111.1, 37.04, 12.35, 4.12, 1.37, and 0 U/mL). The assay protocol, originally produced for human serum, was first optimized for tear analysis by testing different tear dilutions in the sample diluent provided by the assay. Based on the results obtained in positive and control samples, ocular fluid samples were tested at 1:10 dilution. Each sample was assessed in duplicate. Briefly, 100 µL of positive control or tear sample was added to each well and incubated for 1 h at room temperature with gentle shaking. Plates were then washed 4 times with wash buffer (provided with the kit), and 100 µL of previously prepared Biotinylated Anti-Human IgA Antibody was added to each well and incubated for 30 min at room temperature with gentle shaking. After repeating the washing steps, 100 µL of prepared HRP-Streptavidin solution was added to wells, and the plate was further incubated for 30 min at room temperature with gentle shaking. Washing steps were repeated, and 100 µL of TMB One-Step Substrate Reagent was added to each well. The plate was incubated for 15 min at room temperature in the dark, then 50 µL of stop solution was added to each well, and the optical density (OD) developed in each well was immediately read at 450 nm of wavelength. A superimposable plate coated with albumin was assayed in parallel as a negative control. After reading at 450 nm, the OD values of all wells of the albumin-coated plate were subtracted from the values detected in the S1-RBD-coated plate, including positive control and samples. The background-subtracted OD values for each set of duplicate positive controls and samples were used to calculate the mean absorbance values, and then, the average OD detected in the well containing 0 U/mL positive control was further subtracted. The calibration curve was plotted on a log-log scale with positive control concentrations (U/mL) on the x-axis and correspondent OD values on the y-axis. IgA levels were expressed as U/mL and quantified based on the calibration curve: following the manufacturer’s instruction, unknown samples were considered positive for OD values greater than 21.4 U/mL and negative for values less than 21.4 U/mL

Sensitivity, specificity, and accuracy of the assay were, respectively, 76.61%, 97.87%, and 91.38%, as declared by the manufacturer.

### 2.4. Statistics 

The Fisher’s exact test was used to compare the frequency of non-numerical epidemiological parameters (gender, symptom severity) in vaccinated and non-vaccinated groups. ANOVA, Kruskal–Wallis, Mann–Whitney, and unpaired Student’s *t*-test were used to compare numerical parameters, including sIgA antibody frequency of detection and titer, in the two groups and sub-groups. The correlation between presence/amount of ocular sIgA and patients’ variables was calculated by linear regression. Values of *p* ≤ 0.05 were considered statistically significant.

## 3. Results

### 3.1. Features of Enrolled COVID-19 Patients 

Seventy-seven patients with SARS-CoV-2 infection were recruited at the University-Hospital of Ferrara (Italy). All recruited subjects tested positive for the SARS-CoV-2 genome in nasopharyngeal/oropharyngeal swabs (NPS/OPS) detected by specific RT-PCR analysis. As for the virus variants present at the time of enrollment, for all those specimens collected between February and March 2022, the variants that mostly circulated in Italy were Delta (lineage B.1.617.2) and Omicron (lineage B.1. 1.529); from April 2022 onward, the SARS-CoV-2 variant that has circulated to date and supplanted Delta was Omicron (lineages BA.4 and BA.5).

Table 1 summarizes the epidemiological and clinical characteristics of the enrolled subjects. Overall, 25/77 enrolled subjects were asymptomatic at the time of enrollment, while the most frequent COVID-19 clinical manifestations included respiratory symptoms such as interstitial pneumonia, mild respiratory failure, pleural effusion, and basal pneumonia. Only 2 out of the total 77 recruited patients showed ocular symptoms (burning).

The group of vaccinated COVID-19 patients included a total of 63 subjects with a mean age of 75.9 years (range 22–96), including 31 males and 32 females. Overall, 1 subject out of 63 (1.6%) showed ocular symptoms (burning), and 7/63 (11.1%) had ocular comorbidities at the time of enrollment. The mean time between first observation and enrollment was 10.3 ± 8.7 days. COVID-19 signs in the SARS-CoV-2-vaccinated cohort ranged from undetectable (asymptomatic subjects, 22/63, 34.9%) or mild (pauci-symptomatic, 7/63, 11.1%) to specific pulmonary symptoms including pneumonia with variable severity (34/63, 53.9%). Steroid therapy was assumed by 30 out of 63 patients (47.6%). The concentration of the inflammation marker C-reactive protein (CRP) in the blood was 3.31 ± 4.49 mg/dL. Mean blood lymphocyte count in vaccinated cohort was 1.72 ± 1.32 x10^3^/µL.

The group of non-vaccinated COVID-19 patients included a total of 14 individuals, comprising 4 males and 10 females, with a mean age of 66.6 years (range 35–90). Only one subject showed ocular symptoms and eye comorbidity (7.1%), and the time between the first observation and enrollment corresponded to 9.2 ± 7.3 days. Non-vaccinated COVID-19 patients were asymptomatic in 3/14 cases (21.4%), pauci-symptomatic in 1/14 cases (7%), and specifically displaying pulmonary signs including pneumonia in 10/14 cases (71.4%). Ten out of the fourteen enrolled subjects were treated with steroid therapy (71.4%). Mean CRP blood concentration was 2.93 ± 4.67 mg/dL, and mean blood lymphocyte count was 1.99 ± 1.56 × 10^3^/µL.

Table 2 reports the mean values referred to the epidemiological features of the patients enrolled in the two cohorts, evidencing the statistical significance of differences for the analyzed parameters in the two cohorts. In detail, no significant divergence was detected in any of the analyzed parameters, likely due to the low number of enrolled subjects in the two groups. Of note, however, the *p*-value originating from the comparison of symptoms between groups was close to significance for the frequency of asymptomatic and pauci-symptomatic individuals in the vaccinated groups compared to the non-vaccinated one, as expected. 

In Table 3, the features of enrolled subjects subgrouped according to symptoms severity are also shown. No statistically significant differences were highlighted, likely due to the low numbers of subjects in the groups. 

### 3.2. sIgA Analysis in Conjunctival Fluid

**Figure 1:**
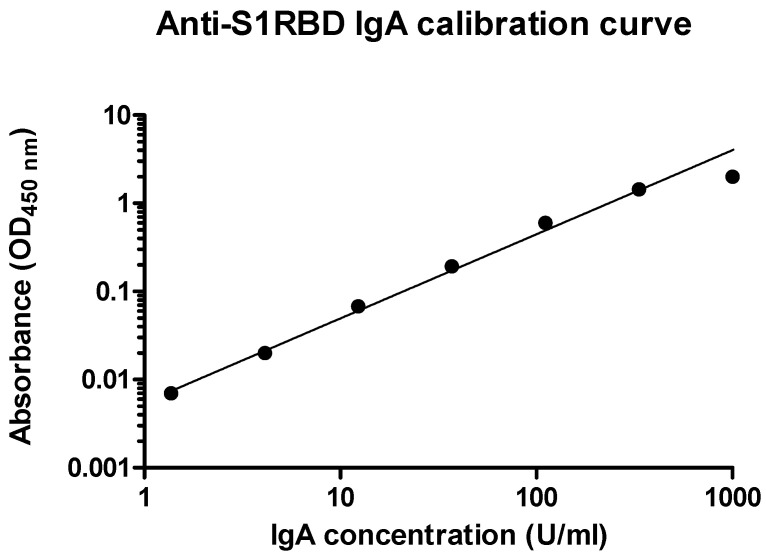
Anti-S1RBD IgA calibration curve used in ELISA assays. The standard curve was obtained by serial dilution of the positive control provided with the kit. Mean OD values of duplicate samples are reported. All values are expressed in a Log/Log graph.

Conjunctival fluid specimens were collected from all recruited subjects by Schirmer test strip from both eyes at 0 to 35 days since the first SARS-CoV-2-positive NPS/OPS swab and consequent hospitalization. Tears samples were immediately refrigerated and processed within 2 h by vortexing and centrifugation. The collected supernatant was then stored at –80 °C until use. Collected samples were tested for the presence of anti-SARS-CoV-2 sIgA by using a quantitative ELISA assay specifically recognizing and quantifying IgA antibodies directed against the RBD portion of the viral spike 1 (S1) protein. The assay protocol was first optimized for conjunctival fluid evaluation, and based on the preliminary results, a 1:10 dilution was chosen for tears evaluation. sIgA levels were expressed as U/mL, and the positivity threshold was set at >21.4 U/mL, as indicated by the manufacturer’s instructions. The standard calibration curve obtained by serial dilution of the positive control provided with the ELISA kit is depicted in Figure 1.

**Figure 2:**
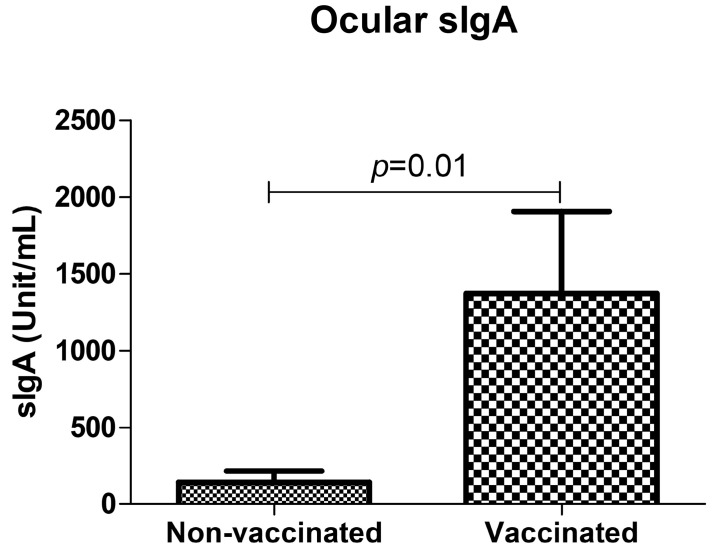
Anti-SARS-CoV-2 ocular sIgA in COVID-19 patients with and without previous vaccination. Ocular IgA values were calculated following manufacturer’s instructions and expressed as Unit/mL. The *p*-value was calculated by the unpaired *t*-test.

By this method, anti-SARS-CoV-2 sIgA antibodies were detected in the tears of 44/63 (69.8%) vaccinated individuals with COVID-19 disease. In those subjects, the ocular sIgA concentrations showed a mean value corresponding to 1372.3 U/mL (range 0–23, 294.9 U/mL) (Figure 1). A remarkably lower prevalence and amount of ocular sIgA were observed in the non-vaccinated group of COVID-19 patients. In detail, specific anti-SARS-CoV-2 sIgA were detectable in 8/14 (57.1%) tear samples from the non-vaccinated cohort, and the positive subjects exhibited a mean sIgA concentration corresponding to 143.7 U/mL (range 0–909.0 U/mL). Statistical analysis did not evidence statistical significance in the difference between vaccinated and non-vaccinated individuals in terms of ocular IgA prevalence despite vaccinated subjects showed a higher percentage of positivity but instead highlighted a significant difference in the mean concentration of ocular sIgA, which was remarkably higher in vaccinated subjects (*p* = 0.01) (Figure 2).

**Figure 3:**
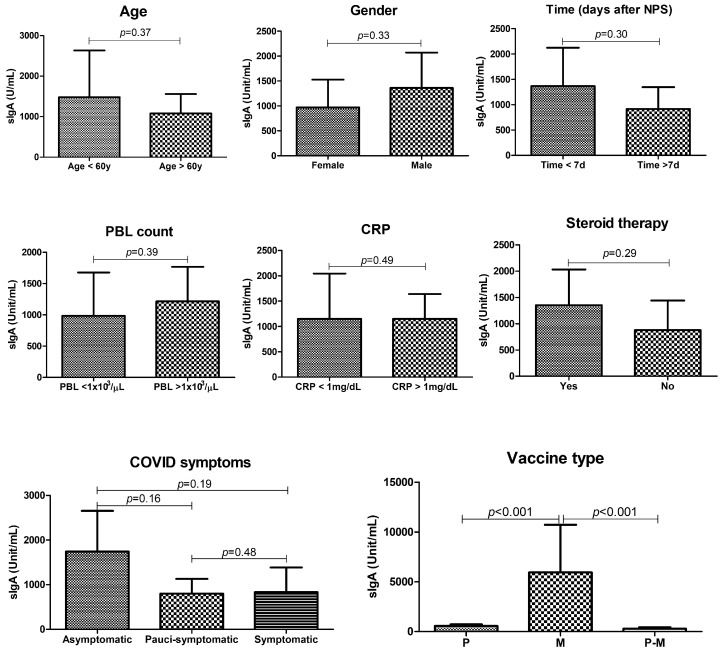
Anti-SARS-CoV-2 ocular sIgA in COVID-19 patients with and without previous vaccination, subgrouped depending on epidemiological and clinical characteristics. Ocular IgA titer is expressed as Unit/mL. The *p*-value was calculated by the Mann–Whitney non-parametric test. PBL, peripheral blood lymphocytes; CRP, C-reactive protein.

Based on the evidenced differences in vaccinated vs. non-vaccinated individuals, we next analyzed the eventual influence of epidemiological and clinical parameters on the production of sIgA at the eye compartment. The statistical analysis after stratification of sub groups depending on the characteristics recorded at enrollment did not evidence significant differences between groups in any of the analyzed parameters: age, gender, days after first positive NPS, PBL count, serum CRP concentration, steroid therapy, and severity of COVID-19 symptoms (Figure 3). By contrast, a clear and significant difference was observed depending on the type of administered vaccine, with the Moderna RNA vaccine showing the highest ability to induce sIgA at the ocular level compared to both Pfizer or combined Moderna and Pfizer vaccinations (Figure 3).

## 4. Discussion

Although it is recognized that mucosal IgA can provide protective immunity against respiratory viruses, the potential role of mucosal IgA in protecting against SARS-CoV-2 infection is still largely unknown. We previously showed an inverse correlation between the titer of mucosal sIgA in saliva and the severity of COVID-19 symptoms, supporting the protective role of an early mucosal IgA response against the progression of the disease [19]. Similar results were also subsequently confirmed in a single-center study on 179 subjects, showing that ocular sIgA levels are directly correlated to immune response to SARS-CoV-2 infection [8]. Mucosal IgA responses have been detected in infected cases in the absence of serum antibody responses, suggesting that mucosal responses may have a key role in the early restriction of virus replication and virus clearance at the site of entry [20]. sIgA have also been found in the milk of 100% SARS-CoV-2 mothers, thus demonstrating that sIgA are produced in response to the infection and are passed to the neonate [21]. Last, mucosal-specific sIgA secretion in saliva and nasal fluid has been correlated with protection in contrast to the systemic antibody titers [22]. Consistent with this, very recent studies have analyzed the ability of anti-SARS-CoV-2 vaccines to boost the mucosal immune response, showing that vaccination can elicit mucosal IgA responses and neutralize IgA at the oral level, which is detectable for over 5 months after vaccination and is enhanced by a subsequent breakthrough infection by SARS-CoV-2 [23,24]. Similarly, a recent study highlighted that mucosal salivary sIgA are induced by mRNA vaccination but are deeply influenced by pre-existing immunity, which boosts sIgA induction after vaccination [25]. In addition, the induction of mucosal IgA response, which was variably induced by SARS-CoV-2 mRNA vaccines, was shown to be associated with protection against subsequent infection, suggesting that COVID-19 vaccines that elicit a durable IgA response may have utility in preventing infection [26]. However, despite the possible virus entry through the conjunctiva and on previous demonstration of local IgA responses at the eye level, no data are currently available on the presence of mucosal responses against the virus in the eye of vaccinated subjects. Based on this and on the observations that a breakthrough infection by SARS-CoV-2 can enhance this response, we analyzed the sIgA presence and amount in vaccinated or non-vaccinated subjects affected by COVID-19.

The results showed that mRNA vaccines can indeed elicit an IgA response at the eye mucosa, which was detected in almost 70% of vaccinated subjects compared to 57% of non-vaccinated individuals, where IgS response was stimulated by infection, as previously reported by us and others [8,11,27]. Of note, the titer of elicited sIgA was remarkably different in the vaccinated vs. non-vaccinated population (1372.3 U/mL vs. 143.7 U/mL, respectively) (*p* = 0.01) and was also significantly different based on the type of administered vaccine. The mRNA Moderna vaccine proved to be the most potent inducer compared to the other vaccine types (Pfizer-BioNTech and Astrazeneca), and this should be considered when a specific mucosal immune response is desirable to increase the level of protection associated with vaccination. Variable induction of mucosal response by diverse SARS-CoV-2 vaccines has been reported previously at the nasal level [28], where the mRNA vaccine Comirnaty was found to elicit a specific nasal IgA response that was not observed with the inactivated whole-virion SARS-CoV-2 vaccine (Coronavac, from Sinovac Life Sciences, Beijing, China). The results of our study support these observations also at the eye level in the conjunctival fluid, suggesting that not all mRNA vaccines are equally able to induce a relevant mucosal response.

As expected, the proportion of asymptomatic/pauci-symptomatic subjects was consistently different in the vaccinated vs. non-vaccinated cohort (46% and 29% of the total, respectively) although the difference did not result statistically significant, likely due to the low number of enrolled subjects. However, the data indicate a correlation between IgA presence at high-titer and milder COVID-19 symptoms, supporting the protective role of the mucosal response against the virus and suggesting a possible meaning also for ocular sIgA. IgA levels may be useful in defense against SARS-CoV-2 replication in the site of entry by neutralizing the virus or aiding in capture by white cells. Of note, the IgA receptor (FcαR or CD89) can be found on the surface of neutrophils, eosinophils, monocytes, some macrophages (including Kupffer cells), and some dendritic cells [10]. It has been recently reported that the development of an-anti SARS-CoV-2 “sterilizing” immunity, able to prevent virus replication and spread, requires neutralizing antibodies at the site of infection, which for respiratory viruses includes mucosal secretion in the upper airways, mouth, and eye [29]. Subjects who had high levels of wild-type spike-specific mucosal IgA were shown to have significantly lower risk of subsequent omicron breakthrough infection compared to those with lower levels, likely due a better control in the initial viral replication [23]. Consistent with this, intranasal vaccines were shown to induce a superior mucosal immunity in animal models, leading to higher protecting immunity against SARS-CoV-2 [30,31,32]. Moreover, broadly neutralizing IgA antibodies in mucosal tissues were reported to neutralize SARS-CoV-2 variants including Omicron [24,33,34]. Last, antibody-dependent enhancement (ADE), a phenomenon capable of facilitating virus entry and pathogenesis, has mainly observed with IgG rather than IgA through the FcγR involvement [35]; thus, a potent mucosal IgA response would be mainly protective.

Major limitations of our study include the low number of enrolled subjects and the lack of parallel analysis of oral and serum antibodies, which may provide important data to understand vaccine-elicited responses and their protection efficiency. These points should be matter for future studies aimed to clarify in detail these aspects.

## 5. Conclusions

The results of our study indicate for the first time the ability of SARS-CoV-2 vaccines to elicit with variable efficiency a specific sIgA response at the eye level, suggesting a correlation between IgA titer and prevention of severe COVID-19 symptoms. Based on these observations, quantitative tear-based sIgA tests may potentially serve as a rapid and easily accessible biomarker for the assessment of the development of a protective mucosal immunity toward SARS-CoV-2.

## Figures and Tables

**Table 1:** Clinical/epidemiological features and sIgA detection in enrolled COVID-19 subjects with or without previous anti-SARS-CoV-2 vaccination.

N°	Age	Gender	Vaccine	COVID-19 Symptoms	Steroid Therapy	Ocular Symptoms	PBL (×10^3^/µL)	CRP (mg/dL)	Time * (Days)
1	67	F	M-M	Interstitial Pneumonia	+	none	2.14	6.38	0
2	60	F	M-M	Asymptomatic	-	none	2.85	0.06	7
3	29	M	P-P	Pulmonary Thromboembolism	+	none	1.13	9.92	26
4	87	F	P-P	Mild Pneumonia	+	none	2.25	2.69	9
5	96	M	P-P	Pauci-Symptomatic	-	none	1.95	1.10	3
6	94	M	P-P	Asymptomatic	-	none	0.34	1.63	7
7	92	F	P-P	Basal Pneumonia	-	none	1.11	1.33	5
8	58	M	P-P	Interstitial Pneumonia	+	none	7.20	1.07	7
9	70	M	P-P	Pauci-Symptomatic	+	none	2.71	2.40	6
10	51	M	P-P	Asymptomatic	+	none	0.33	1.30	34
11	69	F	P-P	Interstitial Pneumonia	+	none	3.98	0.13	5
12	71	M	A-A-M	Asymptomatic	-	none	0.50	10.04	6
13	79	F	A-A-M	Pneumonia		none	1.15	2.37	15
14	88	M	M-M-M	Asymptomatic	-	none	1.50	13.56	4
15	88	F	M-M-M	Interstitial Pneumonia, Respiratory Failure	-	none	0.49	1.51	9
16	22	M	M-M-M	Asymptomatic	-	none	1.20	13.05	1
17	72	F	M-M-M	Pneumonia, Partial Respiratory Failure	+	none	0.66	1.77	11
18	87	F	M-M-P	Interstitial Pneumonia	+	none	0.75	2.47	10
19	91	F	M-M-P	Pauci-symptomatic, Mild Respiratory Failure	+	none	1.74	0.08	15
20	92	F	M-M-P	Interstitial Pneumonia	-	none	0.95	3.22	13
21	87	F	M-M-P	Pauci-symptomatic	-	none	1.35	1.20	30
22	83	F	P-P-M	Interstitial Pneumonia	+	none	1.44	2.53	14
23	69	F	P-P-M	Asymptomatic	+	none	0.23	1.65	22
24	82	M	P-P-M	Pneumonia	+	none	0.96	0.08	14
25	70	M	P-P-M	Pneumonia	+	none	1.82	0.75	9
26	81	F	P-P-M	Pleural Effusion		none	1.39	1.30	33
27	77	M	P-P-M	Asymptomatic	+	none	1.64	0.77	14
28	39	M	P-P-M	Interstitial Pneumonia	+	none	2.40	0.19	3
29	52	M	P-P-M	Asymptomatic	-	none	2.66	0.91	3
30	73	M	P-P-M	Interstitial Pneumonia	+	none	1.54	0.25	16
31	39	M	P-P-M	Interstitial Pneumonia	-	none	1.61	0.45	4
32	80	M	P-P-M	Type 1 Respiratory Failure	-	none	3.67	1.31	13
33	65	M	P-P-M	Asymptomatic	-	none	0.84	20.31	11
34	81	F	P-P-M	Pauci-symptomatic	-	none	3.04	0.86	3
35	84	F	P-P-M	Asymptomatic	-	none	0.94	0.15	3
36	88	F	P-P-M	Asymptomatic	-	none	1.27	5.90	2
37	61	M	P-P-P	Mild Respiratory Failure	+	none	0.58	3.96	5
38	87	F	P-P-P	Interstitial Pneumonia	+	burning	1.25	0.28	16
39	91	F	P-P-P	Asymptomatic	-	none	1.04	6.34	25
40	77	M	P-P-P	Asymptomatic	-	none	1.31	0.30	11
41	89	M	P-P-P	Pneumonia	+	none	6.17	7.46	6
42	49	F	P-P-P	Asymptomatic	-	none	3.20	1.28	5
43	78	F	P-P-P	Asthenia, Dyspnea on Exertion	-	none	2.96	2.10	5
44	86	M	P-P-P	Respiratory Failure	+	none	0.64	17.09	13
45	75	M	P-P-P	Pneumonia	+	none	2.01	1.73	4
46	83	F	P-P-P	Asymptomatic, Mild Desaturation	-	none	4.00	0.04	5
47	83	F	P-P-P	Interstitial Pneumonia	+	none	0.94	13.87	2
48	83	F	P-P-P	Respiratory Failure	+	none	0.68	1.28	6
49	63	M	P-P-P	Asymptomatic	-	none	0.52	0.09	5
50	86	F	P-P-P	Asymptomatic	-	none	0.78	8.33	6
51	85	F	P-P-P	Asymptomatic	-	none	1.96	1.51	3
52	93	M	P-P-P	Pauci-symptomatic	-	none	1.37	1.02	12
53	86	M	P-P-P	Bilateral Bronchopneumonia	-	none	0.48	6.76	35
54	88	M	P-P-P	Pneumonia, Partial Respiratory Failure	+	none	0.73	1.48	2
55	87	F	P-P-P	Fever, Cough, Dyspnea	+	none	1.87	1.98	5
56	84	F	P-P-P	Interstitial Pneumonia	+	none	2.89	1.40	3
57	83	F	P-P-P	Interstitial Pneumonia	+	none	3.28	0.49	8
58	85	M	P-P-P	Asymptomatic	+	none	1.59	9.37	21
59	81	M	P-P-P	Asymptomatic	-	none	0.74	0.92	18
60	83	F	P-P-P	Interstitial Pneumonia	+	none	1.86	3.21	28
61	71	F	P-P-P	Pauci-symptomatic	-	none	1.20	1.07	3
62	74	M	P-P-P	Persistent Cough, Respiratory Failure	-	none	2.99	0.61	3
63	79	M	P-P-P	Asymptomatic	-	none	1.04	0.02	7
64	71	F	-	Pulmonary Thromboembolism	+	no	0.62	17.32	3
65	46	M	-	Fever, Cough, Dyspnea	+	no	1.99	1.63	14
66	61	M	-	Interstitial Pneumonia	+	no	1.22	1.98	13
67	47	M	-	Interstitial Pneumonia	+	no	2.50	0.18	26
68	36	F	-	Pauci-symptomatic, Pulmonary Embolism	-	burning	2.85	0.10	2
69	83	F	-	Asymptomatic	+	no	1.18	8.75	5
70	35	F	-	Mild Respiratory Failure	+	no	2.51	2.49	18
71	75	F	-	Fever, Cough, Dyspnea	-	no	3.57	1.85	7
72	68	F	-	Interstitial Pneumonia	+	no	2.27	1.00	10
73	71	F	-	Pulmonary Lymphoma	+	no	0.03	2.80	6
74	71	F	-	Asymptomatic	-	no	0.31	0.63	1
75	90	M	-	Interstitial Pneumonia	+	no	1.23	1.33	10
76	89	F	-	Asymptomatic	-	no	0.93	0.17	1
77	89	F	-	Interstitial Pneumonia	+	no	1.07	0.73	9

* Time expressed as number of days between the first positive nasopharyngeal/oropharyngeal swab (NPS/OPS) and the Schirmer test. PBL, peripheral blood lymphocytes. Vaccines: M, Moderna; P, Pfizer-Biotech; A, AstraZeneca.

**Table 2:** Epidemiological features of the enrolled cohorts.

Parameters	Vaccinated (*n* = 63)	Non-Vaccinated (*n* = 14)	*p*-Value
Age	75.9 ± 15.8	66.6 ± 19.1	0.06 (n.s.)
Gender (male)	31 (49.2%)	4 (28.6%)	0.23 (n.s.)
Ocular symptoms	1/63 (1.6%)	1/14 (7.1%)	0.33 (n.s.)
Time (days)	10.3 ± 8.7	9.2 ± 7.3	0.68 (n.s.)
CRP (mg/dL)	3.31 ± 4.49	2.93 ± 4.67	0.77 (n.s.)
PBL (×10^3^/µL)	1.72 ± 1.32	1.99 ± 1.56	0.70 (n.s.)
Steroid therapy	30/63 (47.6%)	10/14 (71.4%)	0.14 (n.s.)
COVID-19 signs:			
*Asymptomatic + Pauci-symptomatic*	29/63 (46%)	4/14 (29%)	0.06 (n.s.)
*Symptomatic*	34/63 (53.9%)	10/14 (71.4%)	0.17 (n.s.)

**Table 3:** Features of the enrolled vaccinated and non-vaccinated subjects, separated according to symptoms’ severity into asymptomatic/pauci-symptomatic and symptomatic subgroups.

Parameters	Asymptomatic/Pauci-symptomatic (*n* = 33)			Symptomatic (*n* = 44)		
	Vaccinated (n = 29)	Non-vaccinated (n = 4)	*p*-value	Vaccinated (n = 34)	Non-vaccinated (n = 10)	*p*-value
Age	75.48 ± 16.7	69.8 ± 23.7	0.50 (n.s.)	76.3 ± 15.3	65.3 ± 18.2	0.06 (n.s.)
Gender (male)	16/29 (55.2%)	0/4 (0%)	0.14 (n.s.)	15/34 (44.1%)	4/10 (40%)	0.22 (n.s.)
Ocular symptoms	0/29 (0%)	1/4 (25%)	0.08 (n.s.)	1/34 (2.9%)	0/10 (0%)	0.34 (n.s.)
Time (days)	10.1 ± 8.9	2.3 ± 1.9	0.09 (n.s.)	10.5 ± 8.7	11.6 ± 6.6	0.71 (n.s.)
CRP (mg/dL)	3.63 ± 5.15	2.41 ± 4.23	0.65 (n.s.)	3.04 ± 3.90	3.13 ± 5.05	0.95 (n.s.)
PBL (×10^3^/µL)	1.48 ± 0.97	1.32 ± 1.08	0.76 (n.s.)	1.89 ± 1.56	1.71 ± 1.05	0.71 (n.s.)
Steroid therapy	6/29 (20.7%)	1/4 (25%)	0.84 (n.s.)	24/34 (70.6%)	9/10 (90%)	0.22 (n.s.)

## Data Availability

All data generated by the study are included in the manuscript.
